# Supplementary material for: Identification of Differential Circular RNA Expression Profiles and Functional Networks in Human Macrophages Induced by Virulent and Avirulent Mycobacterium tuberculosis Strains
Source: Int J Mol Sci. 2023 Dec 16;24(24):17561. doi: 10.3390/ijms242417561 (PMC10744075; doi:10.3390/ijms242417561)
Supplement: Supplementary file 1 [file ijms-24-17561-s001.zip › Figure S1-Identification of differential CircularRNA expression profiles and functional networks in human macrophages induced by virulent and avirulent Mycobacterium tuberculos.pdf]

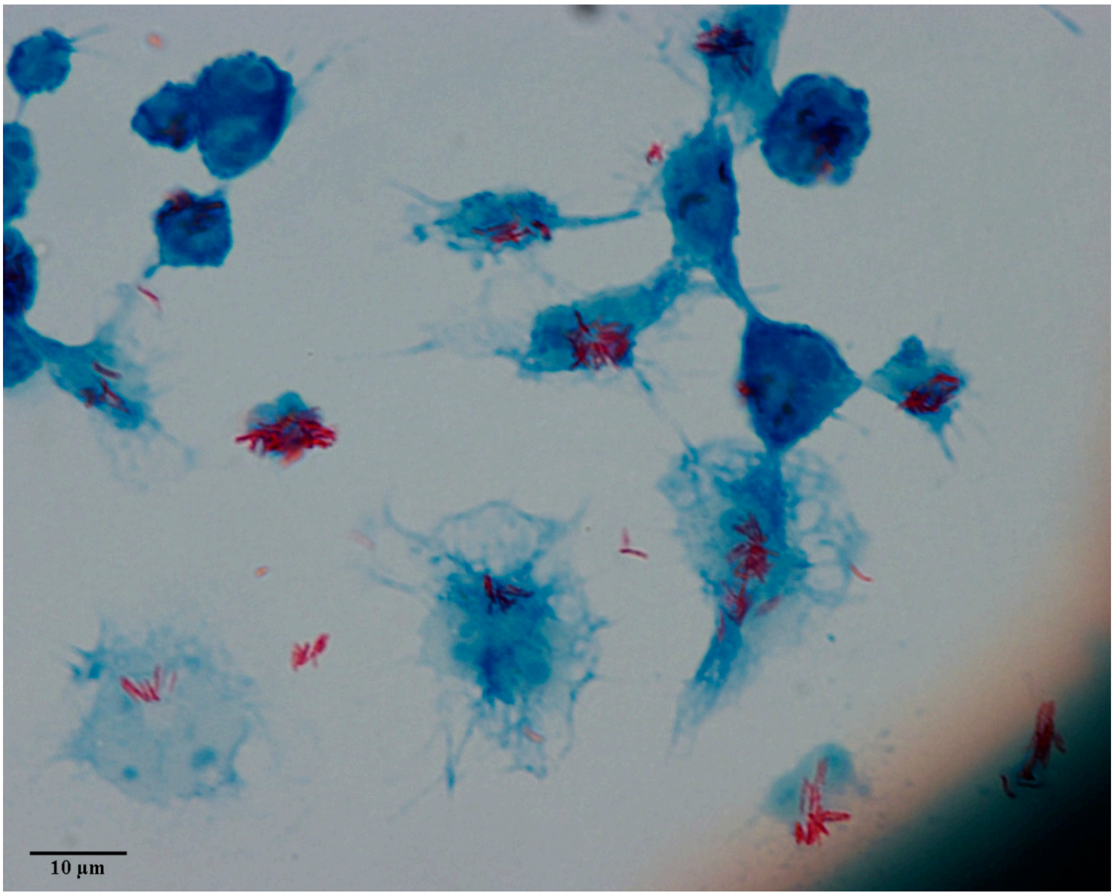

Figure S1: Detection of the quantity of intracellular *M.tb* in THP-1 cells by Acid-fast staining. Red indicated the intracellular *M.tb*. Blue indicated the THP-1 cells. Scale bars, 10 μm.
